# Effect of extracorporeal cytokine removal on vascular barrier function in a septic shock patient

**DOI:** 10.1186/s40560-017-0208-1

**Published:** 2017-01-21

**Authors:** Sascha David, Kristina Thamm, Bernhard M. W. Schmidt, Christine S. Falk, Jan T. Kielstein

**Affiliations:** 1 Department of Medicine, Division of Nephrology & Hypertension, Hannover Medical School, Carl-Neuberg-Str. 1, 30625 Hannover, Germany; 2 Institute of Transplant Immunology, IFB-Tx, Hannover Medical School, Carl-Neuberg-Str. 1, 30625 Hannover, Germany

**Keywords:** Sepsis, Vascular leakage, Endothelial permeability, Cytokines, Extracorporeal removal

## Abstract

**Background:**

Sepsis and septic shock are major healthcare problems, affecting millions of individuals around the world each year. Pathophysiologically, septic multiple organ dysfunction (MOD) is a life-threatening condition caused by an overwhelming systemic inflammatory response of the host’s organism to an infection. We experimentally tested if high circulating cytokine levels might increase vascular permeability—a critical hallmark of the disease—and if this phenomenon can be reversed by therapeutic cytokine removal (CytoSorb®) in an exemplary patient.

**Case presentation:**

A 32-year-old Caucasian female presented with septic shock and accompanying acute kidney injury (Sequential Organ Failure Assessment (SOFA) = 18) to our ICU. In spite of a broad anti-infective regimen, adequate fluid resuscitation, and high doses of inotropics and catecholamines, she remained refractory hypotensive. The extraordinary severity of septic shock suggested an immense overwhelming host response assumingly accompanied by a notable cytokine storm such as known from patients with toxic shock syndrome. Thus, a CytoSorb® filter was added to the dialysis circuit to remove excess shock-perpetuating cytokines. To analyze the endothelial phenotype in vitro before and after extracorporeal cytokine removal, we tested the septic shock patient’s serum on human umbilical vein endothelial cells (HUVECs). The effect on endothelial integrity was assessed both on the morphological (fluorescent immunocytochemistry for VE-cadherin and F-actin) and functional (transendothelial electrical resistance (TER)) level that was recorded in real time with an “electric cell-substrate impedance sensing” (ECIS) system (ibidi). We found (1) severe alterations of cell-cell contacts and the cytoskeletal architecture and (2) profound functional permeability changes, the putative cellular correlate of the clinical vascular leakage syndrome. However, the endothelial barrier was protected from these profound adverse effects when HUVECs were challenged with septic shock serum that was collected after extracorporeal cytokine removal.

**Conclusions:**

Beneficial observations of extracorporeal cytokine removal in septic shock patients might—at least in part—be promoted via protection of vascular barrier function.

## Background

Sepsis is defined as life-threatening organ dysfunction caused by a dysregulated host response to an (often local) infection [1]. Most people do not die from the infection per se but rather from their own overwhelming (inflammatory) response [2]. While the immune system is undoubtedly important in the development of the disease, somewhat less attention has been given to the microvasculature. The endothelium pervades every organ and is responsible for a variety of physiological functions that can be altered in sepsis [3]. The net result is that the septic endothelium presents a pro-coagulant, pro-adhesive surface, fails to produce its usual profile of vasoconstrictive and vasodilatory compounds, and suffers a loss of normal barrier function. Of these changes, increased vascular permeability may be particularly important because it gives rise to hypovolemia and contributes to hemoconcentration, stasis of blood flow, and shock. Thus, systemic vascular changes have severe consequences for organ function and barrier breakdown directly contributing to multiple organ dysfunction (MOD) [4].

The potential of cytokines such as TNFα to induce vascular leakage has been reported. Rather than looking at the immune system and the microvasculature separately as two distinct entities, we wanted to further analyze the link between both systems in a clinical meaningful context. To do this, we used serum from an exemplary septic individual before and after extracorporeal cytokine removal and tested its effect on endothelial morphology and function in vitro.

## Case presentation

A 32-year-old Caucasian female with a 4-day history of fever, malaise, and cough was found unconscious and hypoxic by the emergency team. She was successfully resuscitated and after initial treatment at a local hospital transferred to our institution for extracorporeal membrane oxygenation (ECMO) due to influenza pneumonia, which caused respiratory failure and severe ARDS. She also had an abscess of her left breast that grew *Escherichia coli* bacteria. Due to sepsis (peak CRP 222 mg/L; peak procalcitonin 81.2 μg/L) and accompanying acute kidney injury (AKI), the patient required additional organ support by continuous veno-venous hemodialysis (CVVHD). Sequential Organ Failure Assessment (SOFA) score was 18. The patient remained refractory hypotensive despite a broad anti-infective regimen, adequate fluid resuscitation, and high doses of inotropics and catecholamines. The severity of septic shock suggested an immense overwhelming host response assumingly accompanied by a notable cytokine storm such as known from patients with toxic shock syndrome. Longitudinal clinical and laboratory findings are summarized in Table 1.

**Table 1:** Clinical and laboratory findings

Parameter	Pre	+12 h	+24 h (post)
		Cytokine removal	
Hemodynamics			
Heart rate (bpm)	107	120	111
MAP (mmHg)	58	62	70
NA dose (μg/kg/min)	0.40	0.11	0.09
Respiration			
PaO_2_/FiO_2_	65	68	120
PEEP/ΔP	15/20	15/19	15/18
ECMO			
Pump (rpm)	3650	3650	3650
Blood flow (L)	3.6	3.72	3.54
FiO_2_ (%)	100	100	100
Gas flow (L)	4	4	4
Renal function			
Creatinine (μmol/L)	242	–	70
pH	7.26	7.49	7.47
HCO_3−_ (mmol/L)	18	20	21
Lactate (mmol/L)	3.1	1.8	0.9
UO (mL/h) ±2 h	15	0	0
UF (mL) ±12 h	0	−1200	−4900
Lab parameters			
CRP (mg/L)	222	149	187
PCT (μg/L)	81.2	60.4	34.4
INR	1.45	–	1.18
WBC (1/nL)	41.4	–	23.6
PLT (1/nL)	92	–	41
Hb (g/dL)	12.1	–	10.5

*NA* noradrenaline, *MAP* mean arterial pressure, *PEEP* positive end-expiratory pressure, *P* pressure, *UO* urine output, *UF* ultrafitration, *CRP* C-reactive protein, *PCT* procalcitonine, *INR* international normalized ration, *WBC* white blood cells, *PLT* platelets, *Hb* hemoglobin

Additionally, a CytoSorb® filter was added for a single 24-h session to the dialysis circuit to remove excess shock-perpetuating cytokines. After 24 h of treatment, the mean arterial pressure (MAP) could be maintained above 65 mmHg with markedly reduced need for vasopressors, now even allowing the removal of excessive fluids by ultrafiltration. A clinical observation that might be indicative for stabilization of vascular alterations assumed to contribute to the development and maintenance of shock (such as loss of tone and barrier breakdown). Unfortunately, clinical and radiologic signs of severe hypoxic brain injury forced us to switch our therapeutic strategy to comfort care and the patient died the next day.

Cytokine, chemokine, and growth factor concentrations in supernatants in serum from our patient were quantified by the Luminex-based multiplex technique according to the manufacturer’s instructions (Bio-Rad, USA). Efficacy of cytokine removal by extracorporeal CytoSorb® could be confirmed by comparing pre-treatment and 24 h cytokine levels (Table 2). The observation that some cytokines increased during the treatment might be due to a high biosynthesis overreaching the removal rate. Of note, pre- and post-CytoSorb® drug levels of antibiotics yielded a 76% reduction for meropenem (25.5 to 6.4 μg/mL) and a 58% reduction for piperacillin (11.7 to 4.9 μg/mL). Clindamycin was only reduced by 15% comparing pre- and post-adsorber concentration (14.0 to 11.9 μg/mL). Based on this observation, we highly recommend a thorough therapeutic drug monitoring in septic patients when using extracorporeal removal strategies.

**Table 2:** Cytokine, chemokine, and growth factor removal 24 h after Cytosorb® treatment

Parameter	Pre	Post	Rel. reduction (%)
	Cytokine removal (pg/mL)		
IL-1a	643.9	76	−88.2
IL-6	89.9	14.0	−84.4
CXCL8 (IL-8)	68.4	19.9	−70.9
IL-9	12.9	7.2	−44.2
IL-10	80.7	40.0	−49.6
IL-13	7.1	10.8	+51.5
FGF	48.1	21.4	−55.6
GM-CSF	57.5	38.6	−32.9
CXCL10 (IP-10)	24,723.7	2827.5	−88.6
CCL2 (MCP-1)	375.0	45.9	−87.8
CCL4 (MIP-1b)	125.4	39.2	−68.8
PDGF-bb	719.8	245.3	−65.9
RANTES	3912.2	1326.1	−66.1
TNF-a	59.2	24.8	−58.1
VEGF	63.7	19.5	−69.5

*IL* interleukin, *CXCL* C-X-C motif chemokine, *FGF* fibroblast growth factor, *GM-CSF* granulocyte-macrophage colony-stimulating factor, *IP* interferon gamma produced protein, *CCL* C-C motif ligand, *MIP* macrophage inflammatory proteins, *MCP* monocyte chemotactic protein, *PDGF* platelet-derived growth factor, *RANTES* regulated on activation, normal T cell expressed and secreted, *TNF* tumor necrosis factor, *VEGF* vascular endothelial growth factor

**Fig. 1:**
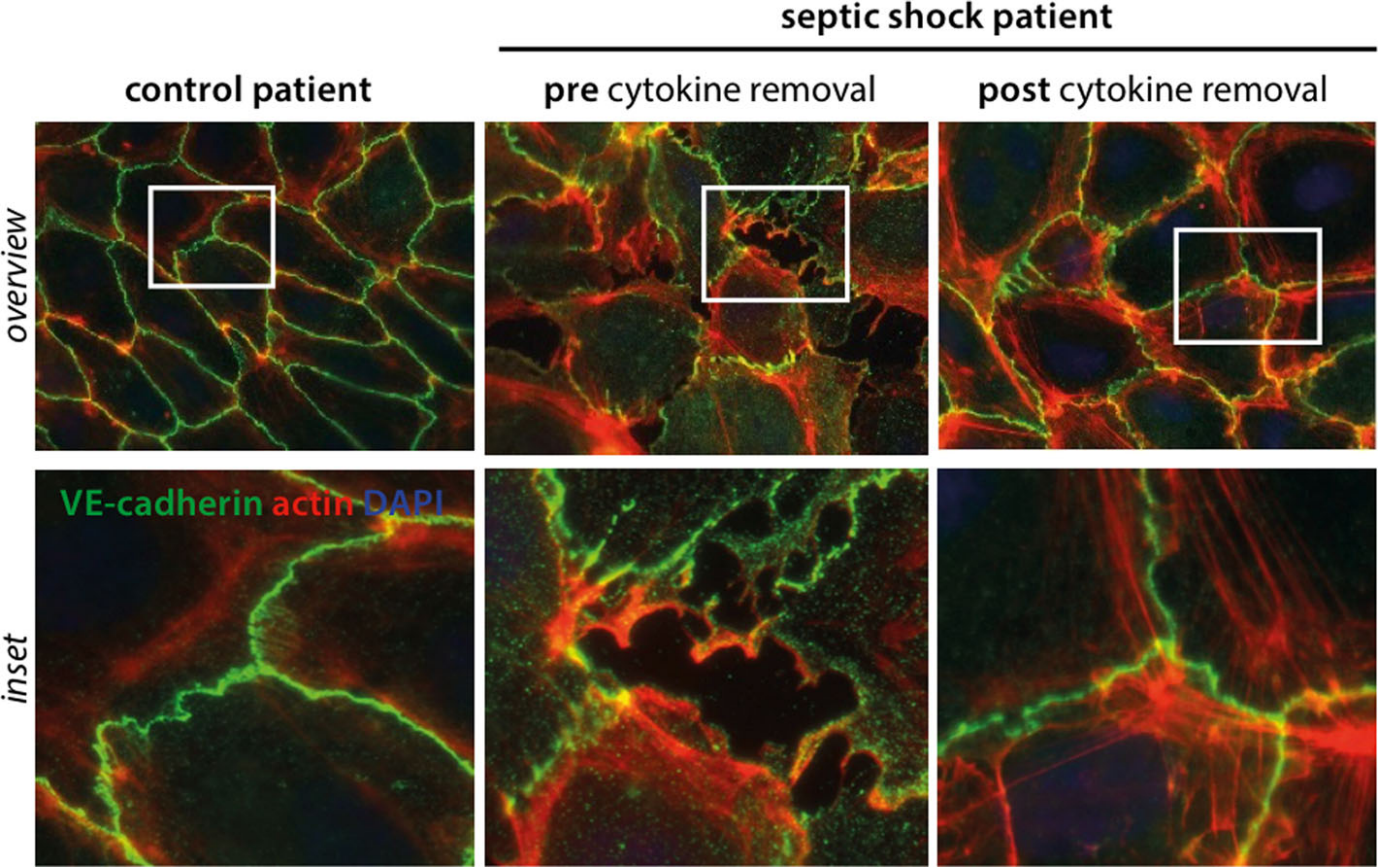
Endothelial phenotype with respect to barrier function. Fluorescence immunocytochemistry staining for vascular endothelial (VE)-cadherin (*green*), F-actin (*red*), was performed on confluent human umbilical vein endothelial cells (HUVECs) as described before [5]. Cells were treated for 30 min with media supplemented with 5% serum from an individual with septic shock before (*2nd row*) and after cytokine removal (*3rd row*); 5% healthy human serum served as a control (*1st row*). *Scale bar* 10 μm

Most notably from the clinical point of view, we found improved hemodynamic stability within the process of cytokine removal (Table 1). In order to experimentally analyze putative cellular effects of the cytokine removal on the vasculature, we used a previously described cellular in vitro system [5]. We therefore collected serum from this patient immediately before and 24 h after cytokine removal. ECs were then challenged with these serial human serum samples for 30 min, and their morphology was investigated by fluorescent immunocytochemistry for the adherence junction protein VE-cadherin (green) and for a major component of the cytoskeleton, i.e., F-actin (red). Of note, intense stress on ECs leads to a polymerization of F-action resulting in the formation of so-called stress fibers. These contraction forces from the cytoskeletal architecture then contribute to the formation of visible gaps between adjacent cells, the cellular correlate for the clinical “vascular leakage syndrome” (Fig. 1, middle panel). When ECs were challenged with serum from the same patient after cytokine removal, the cells showed slightly less formation of stress fibers and were protected from the development of intracellular gaps (Fig. 1, right panel). All together, the endothelial phenotype after cytokine removal was comparable to cells that were stimulated with serum from a healthy control person (Fig. 1, left panel).

**Fig. 2:**
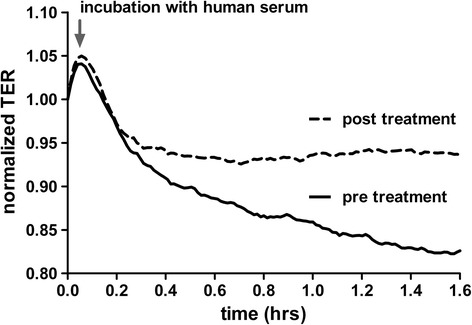
Transendothelial resistance (TER) of HUVECs treated with septic serum. Normalized transendothelial electrical resistance (TER) was longitudinally measured in real time in human umbilical vein endothelial cells (HUVECs) with an electric cell-substrate impedance sensing (ECIS) device. HUVECs were then challenged with either septic serum before cytokine removal (*bold line*) or with serum from the same patient 24 h after cytokine removal (*dotted line*). TER normalization refers to the start point of the experiment for each condition (i.e., 5 min before septic serum incubation)

Next, TER measurements were performed in real time to objectively quantify the functional permeability consequences of these intercellular gaps [6]. ECs were incubated with our patient’s serum before and after cytokine removal analogously to the staining experiment. Serial TERs were recorded every minute with a real-time electric cell-substrate impedance sensing (ECIS) approach in triplicates over 120 min. ECs challenged with serum before cytokine removal showed a rapid drop in mean resistance (consistent with an increase in permeability) (Fig. 2, bold line) whereas serum from the same patient after cytokine removal evidently blunted the permeability response (Fig. 2, dotted line).

## Discussion

Here, we report a case of a severely septic patient with multiple organ failure that was treated with a combination of ECMO and CVVHD. Additional extracorporeal cytokine removal (i.e., CytoSorb®) led to a stabilization of septic shock within hours. Consistent with the hypothesis of a putative interplay between circulating cytokines and altered vascular permeability, we found in cultured ECs that circulating cytokines in septic shock can indeed negatively affect the vascular barrier. One possible approach to remove circulating cytokines could be via modern absorption techniques in particular in those individuals that do require an extracorporeal circuit nevertheless, e.g., for renal replacement or membrane oxygenation therapies. Similar to our patient, the feasibility of combining these extracorporeal devices together with a cytokine removing filter has been exemplary reported in another case in sepsis [7].

Our side observation of lowered antibiotic serum levels is of clinical relevance and highlights the importance of a thorough therapeutic drug monitoring and eventual dose adaptation during modern therapeutic cytokine removal strategies. The same might be true for other protective circulating factors as we have observed with respect to the anti-proliferative cytokine IL-10 in our patient (Table 2). The interaction between the immune system and the microvasculature in the pathogenesis of septic MOD is of particular interest for two main reasons: (1) ECs represent a direct interface between circulating (potentially harmful) cytokines covering virtually all organs and (2) increased permeability with consecutive excessive leakage of intravascular fluids to the interstitial space has increasingly been recognized as a hallmark of MOD and death in sepsis [3, 4]. However, in light with the relatively low absolute cytokine levels before removal, one has to consider that endothelial improvement might be due to off-target removal of other permeability inducing factors.

## Conclusions

Consistent with the concept that circulating cytokines interact with the endothelial surface layer and these cytokines can induce pathological vascular permeability, we observed profound alterations of the endothelial morphology and function when challenged with human septic shock serum in vitro. These cellular changes—that clinically represent the vascular barrier breakdown—were not detectable when serum from the same patient after extracorporeal cytokine removal was used. From this exemplary single patient, we assume that extracorporeal cytokine absorption techniques (such as CytoSorb®) might have protective effects on vascular integrity. No doubt that this report from a single patient is hypothesis generating in nature so that a future systematic study is highly desirable.

## Abbreviations

AKI: Acute kidney injury
CVVHD: Continuous veno-venous hemodialysis
EC: Endothelial cell
ECIS: Electric cell-substrate impedance sensing
ECMO: Extracorporeal membrane oxygenation
HUVEC: Human umbilical vein endothelial cell
MAP: Mean arterial pressure
MOD: Multiple organ dysfunction
SOFA: Sequential organ failure assessment
TER: Transendothelial electrical resistance
TNF: Tumor necrosis factor
VE-cadherin: Vascular endothelial-cadherin
